# Intertwined Roles of Reactive Oxygen Species and Salicylic Acid Signaling Are Crucial for the Plant Response to Biotic Stress

**DOI:** 10.3390/ijms23105568

**Published:** 2022-05-16

**Authors:** Tjaša Lukan, Anna Coll

**Affiliations:** National Institute of Biology, Večna Pot 111, 1000 Ljubljana, Slovenia; anna.coll@nib.si

**Keywords:** plant immune response, reactive oxygen species, salicylic acid, reactive oxygen species–salicylic acid crosstalk, programmed cell death, hypersensitive-response-conferred resistance, RBOH NADPH oxidases, chloroplastic redox state, biosensors, precision transcriptomics

## Abstract

One of the earliest hallmarks of plant immune response is production of reactive oxygen species (ROS) in different subcellular compartments, which regulate plant immunity. A suitable equilibrium, which is crucial to prevent ROS overaccumulation leading to oxidative stress, is maintained by salicylic acid (SA), a chief regulator of ROS. However, ROS not only act downstream of SA signaling, but are also proposed to be a central component of a self-amplifying loop that regulates SA signaling as well as the interaction balance between different phytohormones. The exact role of this crosstalk, the position where SA interferes with ROS signaling and ROS interferes with SA signaling and the outcome of this regulation, depend on the origin of ROS but also on the pathosystem. The precise spatiotemporal regulation of organelle-specific ROS and SA levels determine the effectiveness of pathogen arrest and is therefore crucial for a successful immune response. However, the regulatory interplay behind still remains poorly understood, as up until now, the role of organelle-specific ROS and SA in hypersensitive response (HR)-conferred resistance has mostly been studied by altering the level of a single component. In order to address these aspects, a sophisticated combination of research methods for monitoring the spatiotemporal dynamics of key players and transcriptional activity in plants is needed and will most probably consist of biosensors and precision transcriptomics.

## 1. Reactive Oxygen Species as One of the Earliest Hallmarks of Plant Immune Response

**Figure 1:**
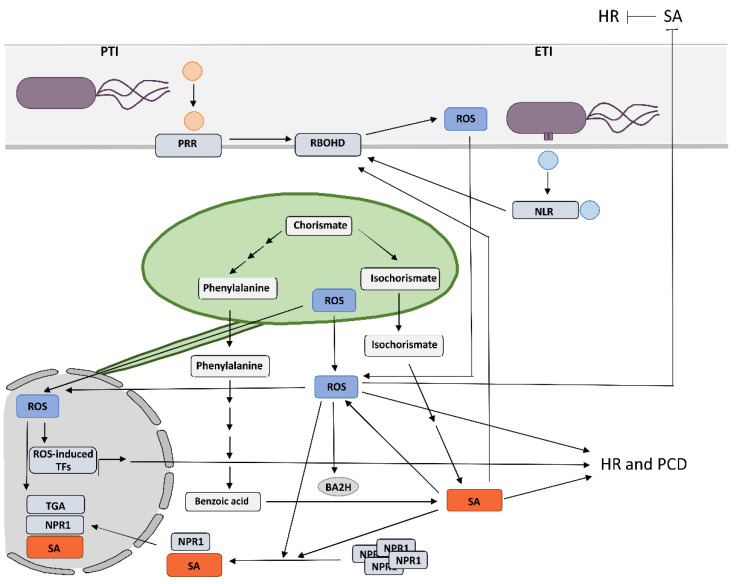
Intertwined roles of reactive oxygen species and salicylic acid signaling in plant response to biotic stress. The scheme represents the crosstalk between salicylic acid (SA) and reactive oxygen species (ROS) signaling. Pathogen-associated molecular-pattern-triggered immunity (PTI) is mediated by cell-surface-localized pattern recognition receptors (PRRs), which recognize conserved microbial- or pathogen-associated molecular patterns (MAPMs or PAMPs) extracellularly. Intracellular-nucleotide-binding domain leucine-rich repeat receptors (NLRs) detect pathogen effector proteins within cells and activate effector-triggered immunity (ETI). Successful ETI often results in hypersensitive response (HR)-conferred resistance and programmed cell death (PCD). The role of RBOHD has been shown in both PTI and ETI. For simplicity, bacterial pathogen induces NLR on the scheme; however, the role of RBOH-generated ROS in HR cell death and/or HR-conferred resistance has also been confirmed for viral, oomycete, and fungal pathosystems. Superoxide produced by NADPH oxidases (RBOH) is spontaneously or by superoxide dis-mutase (SOD) converted to hydrogen peroxide (H_2_O_2_), which crosses plasmalemma via free diffusion or aquaporin-facilitated diffusion to enter the cell. SA is known as a chief regulator of ROS production by regulating RBOH transcription. Plants possess isochorismate synthase and phenylalanine ammonia-lyase pathways to synthesize SA, both starting from chorismate precursor. Benzoic acid is converted into SA by BA2H, which is regulated by ROS. Higher SA level and change of redox state induce monomerization of NPR1, translocation into the nucleus, and NPR1-dependent gene expression through direct interactions with TGA transcription factors. Intercellular ROS inhibits SA accumulation and HR in the adjacent cells. Chloroplastic ROS might, in addition to signaling in HR cell death, also be involved in controlling plant immune responses by reprogramming transcription of genes involved in response to pathogen attack as one of the retrograde signals either directly via stromules or by first entering the cytosol. In the nucleus, control of gene expression depends mainly on the activity of TFs that interact with oxidative-stress-responsive cis-regulatory elements within the gene promoters. The intertwined roles of ROS and SA in immunity are pathosystem- and RBOH-isoform-dependent; however, note that the scheme is simplified. SA: salicylic acid, ROS: reactive oxygen species, RBOHD: respiratory burst oxidase homolog (RBOH) NADPH oxidases D, BA2H: benzoic acid 2-hydroxylase, NPR1: nonexpresser of PR gene 1, TFs: transcription factors.

Plants have evolved sophisticated mechanisms to perceive pathogen attack and trigger an effective immune response through two distinct but inherently intertwined layers of immune response [1] (Figure 1). The first layer, pathogen-associated molecular-pattern-triggered immunity (PTI), is mediated by cell-surface-localized pattern recognition receptors (PRRs), which recognize conserved microbial- or pathogen-associated molecular patterns (MAPMs or PAMPs) extracellularly. The second layer is mediated by intracellular-nucleotide-binding domain leucine-rich repeat receptors (NLRs) [2]. NLRs, also known as R proteins, detect pathogen effector proteins within cells and activate effector-triggered immunity (ETI). Successful ETI often results in hypersensitive response (HR)-conferred resistance. In HR, restriction of pathogens to the infection site is associated with a form of localized programmed cell death (PCD), which is manifested as the formation of necrotic lesions on inoculated leaves [3]. HR-conferred resistance is preceded by a series of biochemical and cellular signals. Whereas the mechanism of R proteins activation is well known, the downstream signaling mechanisms leading to the restriction of the pathogen have been less studied.

Together with the changes in the intracellular calcium levels, one of the earliest hallmarks of HR is rapid and intense production of reactive oxygen species (ROS) in different subcellular compartments [4]. The first phase, transient and with low amplitude, occurs within minutes after infection and is mostly apoplastic, tightly linked to posttranslational activation of plasma membrane respiratory burst oxidase homolog (RBOH) NADPH oxidases and cell wall peroxidases and is attributed to PTI [5]. In Arabidopsis, RBOH activity is regulated through phosphorylation and ubiquitination [6,7,8]. Superoxide produced by NADPH oxidases is spontaneously or by superoxide dismutase (SOD) converted to hydrogen peroxide (H_2_O_2_) [9], which crosses plasmalemma via free diffusion or aquaporin-facilitated diffusion to enter the cell [10,11]. The second phase, sustained and with high amplitude, is initiated a few hours after infection in different compartments, including apoplasts, chloroplasts, mitochondria, and peroxisomes. It requires transcriptional activation of RBOH genes and is associated with the establishment of defense responses and the HR [9,12]. However, it has recently become clear that both layers have been intertwined and share common regulatory mechanisms [13]. The role of RBOHD in PTI is well known in diverse pathosystems [14], while its involvement in HR has been less studied. However, several studies have pointed to the essential role of RBOH-generated ROS also in HR cell death and/or HR-conferred resistance. This includes viral pathosystems [15] such as potato–potato virus Y interaction [16] and one of the most studied viral pathosystems, Nicotiana tabacum—tobacco mosaic virus (TMV) [17,18], bacterial [19,20,21,22,23], oomycete [19,24,25], and fungal pathosystems [23,26,27,28,29]. The results of the above-mentioned studies show that the role of RBOH-generated ROS in immunity is pathosystem- but also RBOH isoform-dependent [14]. Moreover, it has been shown that different RBOH isoforms perform different roles within the same host or in different hosts. Some are involved in cell death induction and/or resistance, while others are not, and some are involved in early, while others in late ROS production; they regulate different signaling pathways in the plant immune response or they function together in the resistance to some pathogens [19,22,28,30,31].

In addition, the effective defense response strongly depends on the action of several plant hormones that ultimately reprogram the transcriptome. Among them, salicylic acid (SA) has been identified as one of the key components of the immune signaling [32]. Its crucial role, not only in ETI but also in PTI and systemic acquired resistance, have been extensively studied [33,34,35].

## 2. Crosstalk between RBOHD-Derived Reactive Oxygen Species and Salicylic Acid in Programmed Cell Death and Resistance

Although ROS regulates plant immunity, biotic stress can cause ROS overaccumulation leading to oxidative stress [36]. Therefore, a suitable equilibrium is crucial for redox homeostasis in the plant. SA is known as a chief regulator of ROS; however, underlying mechanisms are still largely unexplored [37]. In the early 1990s, it was discovered that SA affects ROS production in response to stress [38]. To date, it became clear that SA is required for the restriction of bacterial, oomycete, fungal, and viral pathogens during HR in various pathosystems [3,39,40], including tobacco mosaic virus (TMV) [41,42] and PVY [16,43]. Yet, the exact role of SA and the position where SA interferes with ROS signaling depends on the origin of ROS but also on the pathosystem [44].

One of the main targets of SA to mediate ROS signaling induction is NADPH oxidase RBOH [44]. However, this regulation seems to be pathosystem-dependent. In Arabidopsis, treatment with SA induced ROS production, which resulted from the regulation of the PRRs and was most probably AtRBOHD-dependent [45]. This is also supported by the presence of SA-responsive cis-regulatory elements in the promoters of different RBOHD isoforms in Arabidopsis and rice [46,47]. On the other hand, in potato, SA-induced ROS production is rather related to ETI, as in potato HR-conferred resistance against potato virus Y (PVY), RBOHD was under transcriptional regulation of SA [16]. Similarly, in another study on potato, SA treatment induced RBOHB expression, which was also responsible for the second, ETI-related ROS burst phase [48]. In SA-treated pear fruits, the expression of RBOH was isoform-dependent [49], which is in agreement with the study performed in potato [16].

Under stressful conditions, ROS are not only acting downstream of SA signaling, but were also proposed to be a central component of a self-amplifying loop that regulates SA signaling as well as the interaction balance between different phytohormones [37,50]. RBOHD knockout Arabidopsis mutant plants accumulated higher levels of SA following interaction with pathogens [51]. On the other hand, Chaouch et al. did not detect any difference in SA accumulation in the RBOHD mutant compared with wild-type Arabidopsis [20]. The inconsistency in the results might be connected to tight spatial regulation of SA accumulation as it was shown in potato HR-conferred resistance against potato virus Y (PVY), where accumulation of SA was spatially regulated by RBOHD [16]. Interestingly, however, SA biosynthesis was not controlled by RBOHD-generated ROS in this interaction [16].

There have also been a few indications of the intertwined role of RBOHD and SA in spatial regulation of cell death. Pogany et al. showed that in Arabidopsis, RBOHD triggers death in cells damaged by fungal infection, but simultaneously inhibits death in neighboring cells through the suppression of free SA and ET (ethylene) levels [51]. Consistent with the results of this study, using lsd1 (negative regulator of plant cell death) and rbohd double mutants, Torres et al. showed that in Arabidopsis, AtRBOHD-dependent ROS production at infection sites with elevated levels of SA suppressed SA-dependent HR cell death in neighboring cells [26].

The results of the above-mentioned studies suggest pathosystem-dependent correlation between SA and RBOH-derived ROS in HR cell death and HR-conferred resistance. Different mechanisms in regulating RBOH-dependent ROS production are important for maintaining signaling specificity, while the cross talk with SA signaling provides another layer of regulation.

## 3. Chloroplastic Reactive Oxygen Species Play a Role in the Signaling for Programmed Cell Death and Induce SA-Dependent Transcription of Immune Genes

A growing body of evidence supports a central role of chloroplasts as integrators of environmental signals and key defense organelles, as they host biosynthesis of several key defense-related molecules, including SA and ROS, and are therefore primary sites for the biosynthesis and transmission of pro-defense signals during plant immune responses [12,52,53]. Increases in chloroplastic ROS concentration have been observed in different incompatible plant–pathogen interactions [54], and, in addition, the results of several studies suggest the involvement of chloroplastic ROS in the signaling for and/or execution of HR cell death in HR-conferred resistance [16,55,56,57,58,59,60]. However, its exact role during HR still remains largely elusive. Zurbriggen et al. (2009) suggested that ROS generated in chloroplasts during non-host interaction are essential for the progression of PCD, but do not contribute to the induction of pathogenesis-related genes or other signaling components of the response, including SA signaling [56]. Similarly, Yao and Greenberg did not detect an increase in the expression of genes from SA-mediated signaling in the acd2 mutants that show spontaneous light-dependent PCD and chloroplastic H_2_O_2_ increase [61]. In contrast, Straus et al. suggested that chloroplastic ROS acts as a flexible spatiotemporal integration point leading to opposite SA signaling reactions in infected and surrounding tissue to control the propagation of PCD [57]. Predominance of chloroplast superoxide over H_2_O_2_ drives PCD in infected tissue and RBOHD-regulated restriction of PCD in the surrounding tissue. When the equilibrium is through SA synthesis shifted towards H_2_O_2_ production, this results in runaway PCD [57]. Interestingly, Ochsenbein et al. (2006) also observed that chloroplastic singlet oxygen activates SA-mediated signaling, although SA was not required for a singlet-oxygen-mediated cell death [62]. In potato HR, ROS generated in the chloroplasts around the cell death zone are involved in SA-independent execution of cell death and SA-dependent immune signaling, which are spatially regulated [16,63].

Moreover, recent evidence suggests that chloroplastic ROS might, in addition to signaling in HR cell death, also be involved in controlling plant immune responses by reprogramming transcription of genes involved in response to pathogen attack as one of the retrograde signals [62,64,65,66,67,68,69]. Transmission of pro-defense signals is facilitated by direct connections between chloroplasts and other organelles [70,71]. It has been suggested that stromules, which are extensions in the form of fluid-filled tubules, containing soluble components of the compartments, could facilitate this transmission by enabling more targeted and stronger signal transmission [72]. Stromules are induced by a variety of biotic and abiotic stresses and are involved in retrograde signaling after pathogen invasion, light stress, or movement of chloroplasts within the cell [73,74,75]. However, their direct role in immunity is still largely unresolved [68]. In potato HR-conferred resistance against PVY, stromule formation is induced in close proximity to the cells with oxidized chloroplasts [63]. Since Stonebloom et al. (2012) showed that cell-to-cell transport is negatively regulated by an oxidative shift in chloroplasts, while reductive shift in chloroplasts causes increased cell-to-cell transport, these results could indicate on the potential role of stromules in the signaling for HR-conferred resistance [76]. This is further supported by the fact that stromule formation is induced on the front of virus multiplication zone and is tightly spatiotemporaly regulated by SA signaling [63]. Another type of phenomena that indicate the role of stromules in signaling are the connections of stromules with the plasma membrane, mitochondria, and the nucleus, suggesting that the direct transfer of proteins and metabolites between these organelles and the apoplast could occur [77].

## 4. Reactive Oxygen Species of Different Source and Type Induce Diverse Transcriptional Responses

Distinct subcellular compartments produce different ROS types [78], which could all regulate gene expression [79]. Although each organelle could, in theory, locally manage its own ROS homeostasis, ROS and related signaling intermediates are also involved in interorganellar communication [9,77,80]. For example, chloroplast-derived redox signals could be first integrated in the cytosol or directly transferred to the nucleus, either through physical nucleus–chloroplast interaction or via stromules, in order to control retrograde signaling [81]. Only a small number of proteins targeted to the chloroplast have been also identified in the nucleus to function as retrograde signal transducers in response to biotic and abiotic stresses, some of them being WHIRLY1, the PHD-type transcription factor PTM, and NUCLEAR RECEPTOR INTERACTING PROTEIN 1 (NRIP1) [82]. For example, WHIRLY1 has been proposed to convey the redox status in chloroplasts to the nucleus in a SA-dependent manner [83]. While localization of WHIRLY1 and PTM in both chloroplasts and the nucleus have been shown, the way in which their translocation from chloroplasts to the nucleus occurs is still not known [84,85]. On the other hand, Caplan et al. suggested that after TMV inoculation, the NRIP1 protein is translocated from the chloroplast to the nucleus via stromules [74].

In the nucleus, the control of gene expression depends mainly on the activity of transcription factors (TFs) that interact with oxidative-stress-responsive cis-regulatory elements within the gene promoters. It has been reported that the transcripts generated by increased intracellular H_2_O_2_ levels encode proteins of diverse functional categories including TFs, protein kinases, heat shock proteins, glutathione S-transferases (GSTs), UDP-glucuronosyltransferases (UGTs), and cytochrome P450 monooxygenases (CYPs). Upregulated TFs belong to different stress-related TFs’ families, including WRKY, AP2/ERF, MYB, NAC, heat shock factor (HSF), and ZAT [78]. Interestingly, studies have shown that the transcriptional response to apoplastic ROS produced during oxidative burst has little similarity to the effect of chloroplastic ROS [9]. The roles of individual ROS species from different organelles in transcriptional responses have been studied [86], but since the signaling pathways are connected, multiple effects of different organelle-specific ROS on transcriptional response have to be addressed.

## 5. Tools for Studying Redox State with High Spatiotemporal Resolution with Focus on Cytoplasmic and Chloroplastic Redox State in Hypersensitive Response

**Figure 2:**
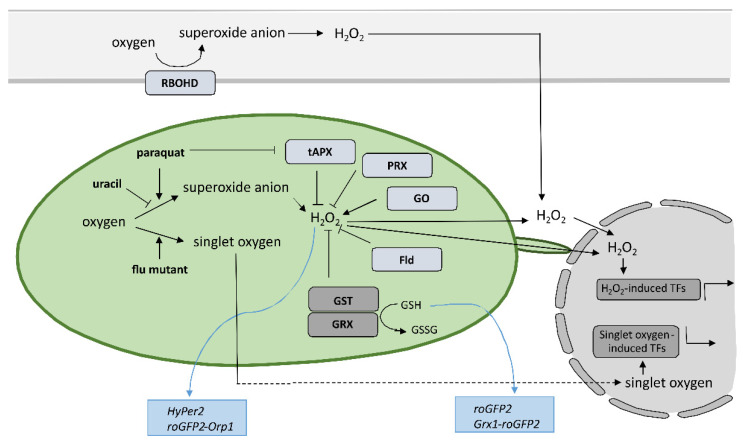
Tools to investigate the role of specific reactive oxygen species produced in chloroplasts and apoplasts. One strategy for studying the role of organelle-specific redox state is by altering reactive oxygen species (ROS) production by using chemicals or mutants (bold) or by altering expression of genes involved in ROS production (light grey). The role of apoplastic ROS could be studied by altering the expression of *RBOHD*. The role of chloroplastic ROS could be studied in transgenic plants with overexpressed *tAPX*, which results in decreased chloroplastic ROS production, or in transgenic plants with estrogen-inducible RNAi-silenced *tAPX* expression, which results in increased chloroplastic ROS production. As PRX similarly as APX reduce H_2_O_2_ accumulation, silencing of *PRX* results in enhanced chloroplastic H_2_O_2_ production. Fld improves the delivery of reducing equivalents to productive pathways of the chloroplast, which in turn restricts chloroplastic ROS production. Paraquat acts by re-directing electrons from photosystem I to oxygen and thereby enhances the production of superoxide in the chloroplasts but also inhibits APX, which leads to accumulation of H_2_O_2_ in treated plants. *Flu* mutant is used to study singlet-oxygen-specific ROS signaling. Uracil, acting as a chloroplast electron transport chain inhibitor, reduces H_2_O_2_ production. Different types of ROS induce specific transcriptional response. Another strategy for studying the role of organelle-specific redox state is by using biosensors (blue). roGFP2 and Grx1-roGFP2 targeted to chloroplasts measure chloroplast redox state, while roGFP2-Orp1 and HyPer-derived probes detect H_2_O_2_. RBOHD: respiratory burst oxidase homolog (RBOH) NADPH oxidases D, tAPX: thylakoidal ascorbate peroxidase, PRX: 2-Cys peroxiredoxin, GO: glycolate oxidase, Fld: flavodoxin, GRX: glutaredoxin, GST: glutathione S-transferase, GSSG: glutathione disulfide, GSH: glutathione.

Despite the new insights that have been brought into the role of redox mechanisms in plant defense response, still one of the major challenges is to monitor local, subcellular, and global ROS dynamics with high selectivity, sensitivity, and spatiotemporal resolution that allow for quantification [87]. Small organic-molecule-based probes have been originally used to measure ROS in plants; however, they have significant limitation as they do not allow for spatiotemporal resolution, since the fluorescence changes as a result of ROS presence are irreversible [88,89]. Nondestructive real-time measuring of redox state in plants with high spatial and temporal resolution is feasible since Jiang et al. reported the use of redox-sensitive green fluorescent protein (roGFP) [90] (Figure 2). Measurement of roGFP fluorescence intensity following excitation with two different wavelengths enables the calculation of the ratio between reduced and oxidized roGFP and thus the determination of the redox state on the cellular or organelle level. The biosensors have been modified by the addition of signal sequences to target them to different subcellular organelles, while two variants, roGFP1 and roGFP2, with different excitation and emission spectra, were developed to allow for optimal selection according to the redox state in the particular organelle. By fusing the peroxisomal targeting peptide sequence SKL, per-roGFP1 and px-roGFP2 were targeted to peroxisomes [91,92]. mt-roGFP1 and mt-roGFP2 are available for measuring the redox state in mitochondria due to the fusion with mitochondrial localization signal peptide from the tobacco b-ATPase [90,92], while er-roGFP2 was constructed by fusing roGFP2 with the endoplasmic reticulum (ER) retention signal peptide HDEL for following the redox state in ER [93]. Finally, cp-GFP2, pt-roGFP2, and chl-roGFP2 were constructed by adding plastid-targeting signal peptide TKTP, coding sequence for RuBisCo small subunit transit peptide, or the first 74 amino acids from PRXa to roGFP2 coding sequence, respectively, for measuring change of redox state in chloroplasts [76,94,95]. Since pt-roGFP is targeted to the chloroplast outer plastid envelope membrane, it also allows for the imaging of stromules formation [73]. The above-mentioned sensors were used in tobacco, Arabidopsis, and potato subjected to different developmental and environmental stresses [73,76,90,91,92,93,94,95,96,97,98,99], some of them also in HR [63,100,101]. roGFP2 expressed in the cytosol senses the redox potential of the cellular glutathione buffer via glutaredoxin (GRX) as a mediator of reversible electron flow between glutathione and roGFP2 [93]. To facilitate specific real-time equilibration between roGFP2 and the glutathione redox couple, fusion constructs with human glutaredoxin 1 (GRX1) Grx1-roGFP2 and roGFP2-Grx2 were generated [102,103,104]. Moreover, two roGFP derivatives, roGFP2-iL and roGFP1-iX, with different midpoint potential and excitation properties, were developed to further extend the range of suitable probes [105]. Another group of genetically encoded sensors that detect H_2_O_2_ levels instead of measuring glutathione redox potential was also developed. roGFP2-Orp1, based on a redox relay between the GPX-like enzyme oxidant receptor peroxidase-1 (Orp1) from *Saccharomyces cerevisiae* and roGFP2 was generated for sensing transient changes in H_2_O_2_ [98]. Another sensor that reports on local alterations in H_2_O_2_ concentrations exploits the H_2_O_2_-sensitive bacterial transcription factor OxyR for its response and was named HyPer [106,107]. The above-mentioned types of sensors were also used to follow redox potential and H_2_O_2_ in the cytoplasm during HR [108,109,110,111,112,113] or were further upgraded to follow redox potential in different cellular compartments, including chloroplasts [114]. Using HyPer2, Exposito-Rodriguez et al. showed that in photosynthetic *Nicotiana benthamiana* epidermal cells, exposure to high light increased H_2_O_2_ production in chloroplast stroma, cytosol, and nuclei, suggesting direct H_2_O_2_ transfer from chloroplasts to nuclei [115]. Other genetically encoded redox and H_2_O_2_ sensors are reviewed in [87,116]. The most recent ones are biosensor CROST for measurements of the thioredoxin redox state in chloroplasts [117], FRET-based biosensors, and ROS regulated promoter–FP fusions [87,118].

Another strategy for studying the role of organelle-specific redox state is by altering ROS production in the organelle of interest; here, we focus on chloroplasts. Chloroplasts produce various forms of ROS during photosynthesis. One of the most reactive ones is singlet oxygen, which is produced by energy transfer from excited triplet state chlorophyll to the oxygen, mainly in the photosystem II reaction center [119]. The other major source of chloroplastic ROS is Mehler reaction in photosystem I, which reduces oxygen to superoxide anion that is further converted to H_2_O_2_ by thylakoid-bound and stromal superoxide dismutases [120]. H_2_O_2_ is reduced in reactions catalyzed by 2-Cys peroxiredoxin (PRX) and ascorbate peroxidase (APX) [121]. Therefore, the role of chloroplastic ROS could be studied in transgenic plants with overexpressed *thylakoidal ascorbate peroxidase* (*tAPX*), which results in decreased chloroplastic ROS production [61,122] or in transgenic plants with estrogen-inducible RNAi silenced *tAPX* expression, which results in increased chloroplastic ROS production [66]. Inducible silencing of *tAPX* increased H_2_O_2_ production in chloroplasts, which activated SA biosynthesis and SA-inducible gene expression [66]. Interestingly, however, over-expression of *stromal ascorbate peroxidase* (*sAPX*) or treatment with photosynthesis inhibitor DCMU attenuates nuclear H_2_O_2_ accumulation and high-light-responsive gene expression, while *cytosolic ascorbate peroxidase* overexpression has little effect [115]. This was explained by the direct H_2_O_2_ transfer from chloroplasts to nuclei, avoiding the cytosol, which enables photosynthetic control over gene expression [115]. As PRX similarly as APX reduces H_2_O_2_ accumulation, silencing of *PRX* results in enhanced chloroplastic H_2_O_2_ accumulation. By VIGS-induced *PRX* silencing, Ishiga et al. showed that PRX functions as a negative regulator of pathogen-induced cell death in the healthy tissue that surrounds the lesions, while chloroplastic ROS play a role in the cell death initiation [58]. The role of chloroplastic ROS could also be studied by the use of transgenic plants with chloroplast-targeted flavodoxin (Fld) [123]. Fld improves the delivery of reducing equivalents to productive pathways of the chloroplast, which in turn restricts chloroplastic ROS production. The introduction of a Fld in chloroplasts of various plant species resulted in increased tolerance to different biotic and abiotic stresses [56,123,124,125,126,127,128]. Plants overexpressing *glycolate oxidase* (*GO*) are another system for studying the effects of chloroplastic H_2_O_2_ [129]. By exploiting this system, Schmidt et al. showed that H_2_O_2_ dosage in Arabidopsis chloroplasts regulates HR-conferred resistance to hemibiotrophic fungus by the induction of WRKY33 [130]. While the above-mentioned systems modulate chloroplastic H_2_O_2_ accumulation, Arabidopsis *flu* mutant manifest increased singlet oxygen production in chloroplasts upon dark/light shift [131], which leads to induced SA synthesis and suppressed spread of necrotic lesions [62]. ROS production in the chloroplasts can also be enhanced by using inhibitors and redox catalysts. The herbicide methyl viologen (paraquat) acts by re-directing electrons from photosystem I (PSI) to oxygen and thereby enhancing the production of superoxide in the chloroplasts [132]. As it also inhibits APX, this leads to accumulation of H_2_O_2_ in treated plants [133]. To study the role of chloroplast-derived photo-oxidative stress in different cellular components, Ugalde et al. treated Arabidopsis seedlings with methyl viologen and recorded dynamic changes in glutathione redox potential and H_2_O_2_ levels with the genetically encoded biosensors Grx1-roGFP2 and roGFP2-Orp1 targeted to chloroplasts, the cytosol, or mitochondria [114]. Similarly, the role of chloroplastic ROS was studied using uracil, a chloroplast electron transport chain inhibitor, which significantly reduced ROS generation and delayed necrosis appearance in biotic stress [134].

## 6. Conclusions

The results of the above-mentioned studies suggest that the precise spatiotemporal regulation of key players, including organelle-specific ROS and SA levels, determines the effectiveness of pathogen arrest and is therefore crucial for a successful immune response. The change of SA and ROS levels and other key players alter the rate of cell-to-cell and systemic pathogen spread, rate of cell death induction, and spatial transcriptional response, leading to susceptibility or resistance. We suggest that only a coordinated and intertwined action of all main components enable effective immune response. However, the specific interactions between them and the regulatory interplay behind still remain poorly understood, as up until now, the role of organelle-specific ROS and SA in HR-conferred resistance has only been studied by altering the level of a single component. In order to address these aspects, a sophisticated combination of research methods for monitoring the spatiotemporal dynamics of key players and transcriptional activity in plants is needed. The precise sampling of tissue sections surrounding the HR-PCD, with spatial resolution and suitable for transcriptomics analyses [16], in combination with the use of biosensors [135], could enable identification of novel key players and could unravel the interconnectivity of immune signaling components. Such an approach could therefore present a step forward in studying the resistance response.
